# Epidemiology and Management of Postoperative Infections in Hepatectomy and Pancreaticoduodenectomy

**Published:** 2025-12-19

**Authors:** Angelie Pathak, Hina Patel, Jason Zar, Devendra K Agrawal

**Affiliations:** Department of Translational Research, College of Osteopathic Medicine of the Pacific, Western University of Health Sciences, Pomona, California 91766 USA

**Keywords:** Bile colonization, Biofilm formation, Empiric antibiotic therapy, Hepatobiliary surgery complications, Multidrug-resistant (MDR) infection, Preoperative biliary drainage

## Abstract

Infections remain a major source of postoperative morbidity and mortality following complex bile duct surgeries, namely hepatectomy and pancreaticoduodenectomy. Infection rates remain high—ranging from 24% to 61%—and are influenced by multiple factors, including patient comorbidities, surgical complexity, and perioperative variables such as prolonged operative time, excessive blood loss, and preoperative biliary drainage. Disruption of the biliary barrier due to drainage or stent placement promotes bile stasis, microbial colonization, and biofilm formation, facilitating the emergence of multidrug-resistant (MDR) pathogens such as Escherichia coli, Klebsiella pneumoniae, and Enterococcus species. These resistance mechanisms—such as β-lactamase production, altered membrane permeability, efflux pump activity, and target site modification—make antimicrobial therapy increasingly complex, prolonging recovery, hospitalization, and healthcare costs. Diagnosis is often challenging and relies on a combination of clinical assessment, inflammatory markers (including leukocytosis, C-reactive protein, and procalcitonin), and microbiological cultures from bile, wound, or drainage fluid. Once infection is confirmed, management requires early recognition, empirical broad-spectrum antibiotic coverage, and subsequent de-escalation based on susceptibility testing. To prevent recurrence, effective source control through interventional drainage or reoperation is essential. In select cases, antifungal therapy may be indicated, particularly in the presence of Candida coinfection associated with biliary interventions or prolonged antibiotic exposure. Preventive strategies—including optimized perioperative care, tailored antibiotic prophylaxis, nutritional optimization, and minimally invasive surgical approaches—are vital to reduce infectious risk. Future efforts should focus on refining risk stratification, advancing rapid diagnostic methods, and developing evidence-based protocols to address the growing challenge of MDR infections and improve postoperative outcomes in hepatobiliary surgery.

## Introduction

Bile duct surgeries encompass a range of procedures, from routine operations to highly complex interventions. This review primarily focuses on hepatectomy and pancreaticoduodenectomy, with particular emphasis on postoperative infectious outcomes. These surgeries are associated with high infection rates, reported between 24% and 61%, depending on the procedure [1]. Several factors influence these outcomes, including patient characteristics, perioperative variables, and the inherent complexity of the surgery, with procedural complexity being one of the most significant determinants.

## Risk Factors for Postoperative Infection

**Figure 1:**
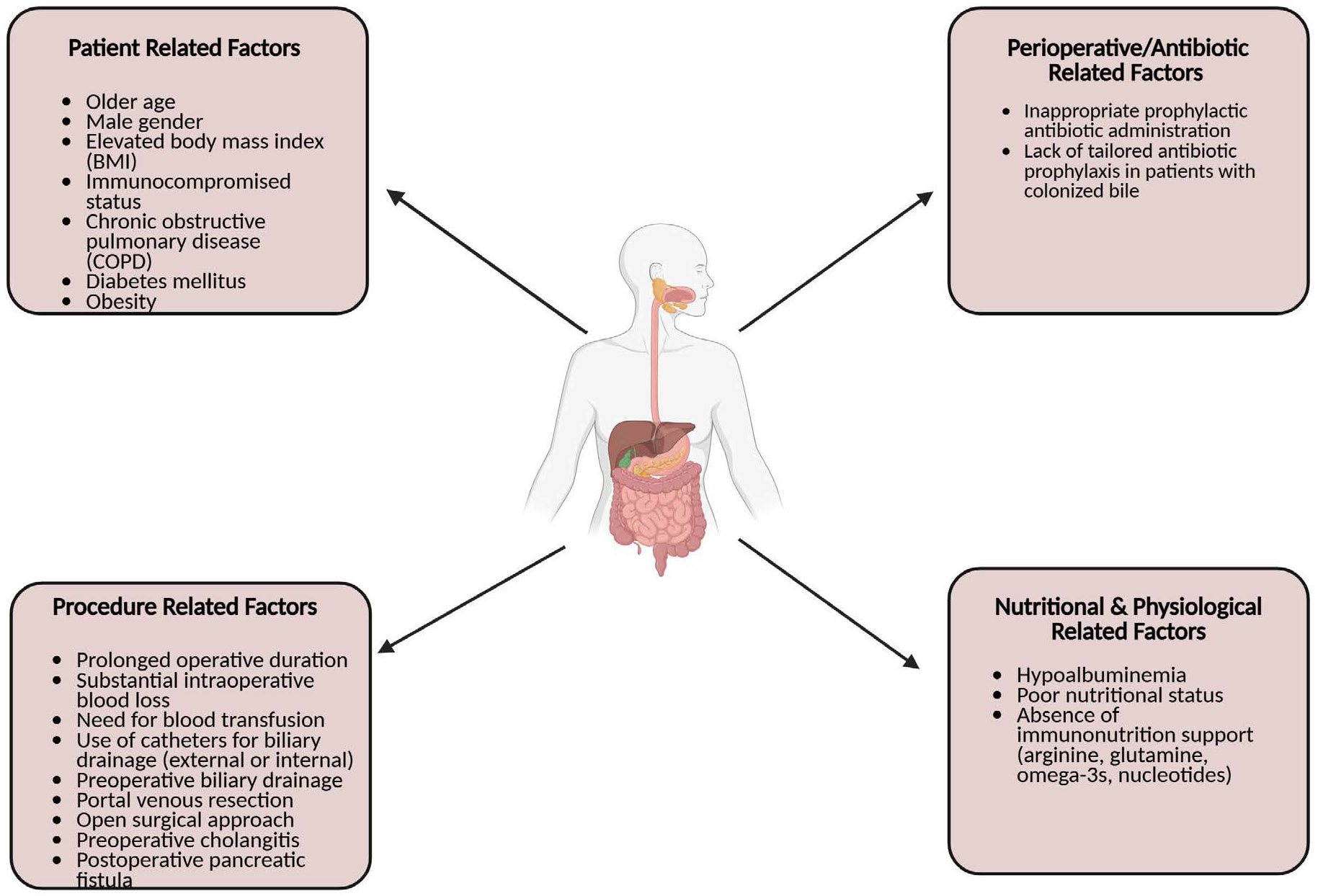
Factors contributing to postoperative infections in gastrointestinal surgery. The diagram illustrates four major categories of risk factors: patient-related factors, procedure-related factors, perioperative/antibiotic-related factors, and nutritional and physiological factors. Together, these elements increase susceptibility to postoperative infectious complications.

Patient-related factors linked to negative postoperative outcomes include older age, male gender, elevated body mass index, immunocompromised status, and comorbidities such as chronic obstructive pulmonary disease (Figure 1). Perioperative and postoperative contributors include prolonged operative duration, intraoperative blood loss, transfusion requirements, and the use of external or internal biliary drainage catheters [2]. Additionally, inappropriate prophylactic antibiotic administration—particularly when failing to target bile-cultured organisms or multidrug-resistant species—can exacerbate adverse outcomes [3,4].

## Findings from Retrospective and Multicenter Studies

Multicenter studies have identified several key risk factors for postoperative infection following hepatectomy and pancreaticoduodenectomy. These include diabetes, obesity, preoperative biliary drainage, prolonged operative time, and open or complex surgical approaches. The highest odds ratios (OR) and infection risks are observed with portal venous resection (OR 5.5) and open approach (OR 4.99) after hepatectomy, and with preoperative cholangitis (OR 10.07) and postoperative pancreatic fistula (OR 6.53) after pancreaticoduodenectomy [5]. These findings highlight that both patient comorbidities and procedure-related factors significantly increase infection risk, emphasizing the importance of targeted perioperative optimization and infection-prevention strategies.

## Modifiable Perioperative Risk Factors

Modifiable factors include nutritional optimization, surgical efficiency, antibiotic stewardship, and drainage management. Monitoring and correcting hypoalbuminemia reduces infection risk, as low albumin is a strong predictor of surgical site infection. In patients with biliary drainage, tailored antibiotic prophylaxis guided by preoperative screening and bile cultures can reduce infection rates and mitigate the effects of colonized bile [6]. Evidence supports the use of preoperative immunonutrition (arginine, glutamine, omega-3 fatty acids, and nucleotides), with administration for 5–7 days shown to reduce infection (OR 0.45–0.62), anastomotic leak (OR 0.67), and hospital stay duration (−1.26 to −1.94 days) [7].

## Comparative Morbidity and Mortality

Hepatectomy with extrahepatic bile duct resection and pancreaticoduodenectomy are particularly prone to infectious complications. In hepatectomy, biliary reconstruction increases the risk of bile leakage and postoperative cholangitis, while pancreaticoduodenectomy is associated with infection due to biliary obstruction and stenting, which predispose the biliary system to microbial contamination.

Globally, outcomes vary widely. After hepatectomy, 90-day mortality ranges from 1.3% for minor resections to 11.4% for extended resections, with major complications (Clavien-Dindo ≥ III) occurring in 22–39% of cases [8]. For pancreaticoduodenectomy, the 90-day mortality is around 5% worldwide but lower (<3%) in high-volume centers. Morbidity remains high (40–68%), with major complications in 18–27% and pancreatic fistula in 10–26%. Increasing procedural complexity, particularly with vascular resections, raises morbidity up to 87% and in-hospital mortality to 18%. Centralization to high-volume centers and standardized perioperative protocols may reduce these risks and improve surgical outcomes globally [9].

## Hepatectomy

Hepatectomy is most often indicated for complex or malignant hepatobiliary diseases, most commonly hepatocellular carcinoma and colorectal liver metastases. These operations are classified as major hepatectomy, involving resection of three or more liver segments, or minor hepatectomy, involving one to two segments [10]. In cases of carcinoma with bile duct involvement, hepatectomy may also include extrahepatic bile duct resection and biliary reconstruction, which increase procedural complexity and the risk of complications such as bile leakage, cholangitis, and surgical site infection [11].

The segmental anatomy of the liver is fundamental in planning and performing hepatectomies. It is defined by the portal triad, which consists of the portal vein, hepatic artery, and bile ducts, allowing for precise resection of diseased tissue while preserving functional parenchyma. This anatomical precision is essential to maintain hepatic function and minimize the risk of postoperative liver failure, particularly in patients undergoing extensive resections. Preoperative imaging plays a critical role in delineating vascular and biliary anatomy, identifying anatomical variations, and guiding the surgical approach [12].

Preoperative considerations for hepatectomy and pancreaticoduodenectomy first includes assessing liver function (Child-Pugh and MELD scores), future liver remnant (FLR) volume, and the presence of portal hypertension. The American Association for the Study of Liver Diseases (AASLD) provides recommendations for resection only in certain cases these include patients with compensated cirrhosis (Child-Pugh A), absence of clinically significant portal hypertension, and an adequate FLR (>30% in noncirrhotics, >40% in cirrhotic [13]. Additionally accounting for patient-related factors such as advanced age, comorbidities, elevated ASA score, and underlying liver disease can all lead to increase perioperative risk [14]. In evaluating postoperative outcomes, these are primarily influenced by procedural complexity and perioperative variables such as prolonged operative time, increased blood loss, and need for transfusion. Complication rates range from 24% to 61%, with the highest rates seen in complex resections and high-risk patients [15].

## Pancreaticoduodenectomy (Whipple Procedure)

It is key to assess overall liver function and the amount of healthy tissue that will remain post resection. Use of the Child-Pugh, MELD, and ALBI scores, help evaluate liver reserve and the risk of decompensation. In other cases, measures such as indocyanine green clearance or liver stiffness measurements are used to gauge functional capacity. The future liver remnant (FLR) is measured using CT or MRI imaging. There can be different signs pointing to portal hypertension which include low platelets, ascites, or varices which all can increase the risk of postoperative complications [16]. Jaundice patients may require preoperative biliary drainage, particularly in cases of infection, poor nutrition, or small future liver remnant is present. Endoscopic drainage is generally preferred over percutaneous methods because it carries fewer complications [17].

## Imaging, Staging, and Tumor Evaluation

Detailed imaging is essential to plan both hepatic and pancreatic operations. High-resolution CT or MRI scans help define the liver’s segmental anatomy, identify any vascular or biliary variations, and detect hidden metastases. When needed, endoscopic ultrasound, ERCP, or PET/CT can provide additional information about tumor involvement and spread [18]. Blood tests measuring tumor markers such as CA 19-9, CEA, and CA125 can help evaluate cancer behavior and the likelihood of advanced disease. Elevated levels often suggest a higher chance of metastatic spread and may influence the decision to proceed with surgery or consider alternative treatments [19].

## Operative Considerations: Hepatectomy and Pancreaticoduodenectomy

Hepatectomy is most often performed for complex or malignant liver diseases, including hepatocellular carcinoma and colorectal liver metastases. Resections can be minor, involving one or two segments, or major, involving three or more. In advanced cases, the surgery may include bile duct resection and reconstruction, which increases the risk of complications such as bile leaks and infections [20]. The Whipple procedure, or pancreaticoduodenectomy, is typically done for cancers involving the pancreatic head or bile duct. This operation removes the head of the pancreas, gallbladder, distal bile duct, and part of the small intestine, followed by reconstruction to restore digestive continuity. Because of the close relationship between the pancreas, bile duct, and major blood vessels like the portal vein and superior mesenteric vessels, the procedure is technically challenging [21]. The uncinate process, which curves around these vessels, often complicates dissection even further. Due to its complexity and the presence of multiple anastomoses, the Whipple carries a high risk of infection, bile leak, and pancreatic fistula—especially in patients with preoperative stenting or biliary obstruction [22].

## Bile Contamination and Microbial Colonization

Bile contamination—the presence of microorganisms in the biliary tract—is a key factor in postoperative infections. Under normal conditions, bile is sterile; however, surgical manipulation and preoperative biliary drainage significantly alter this environment.

**Figure 2:**
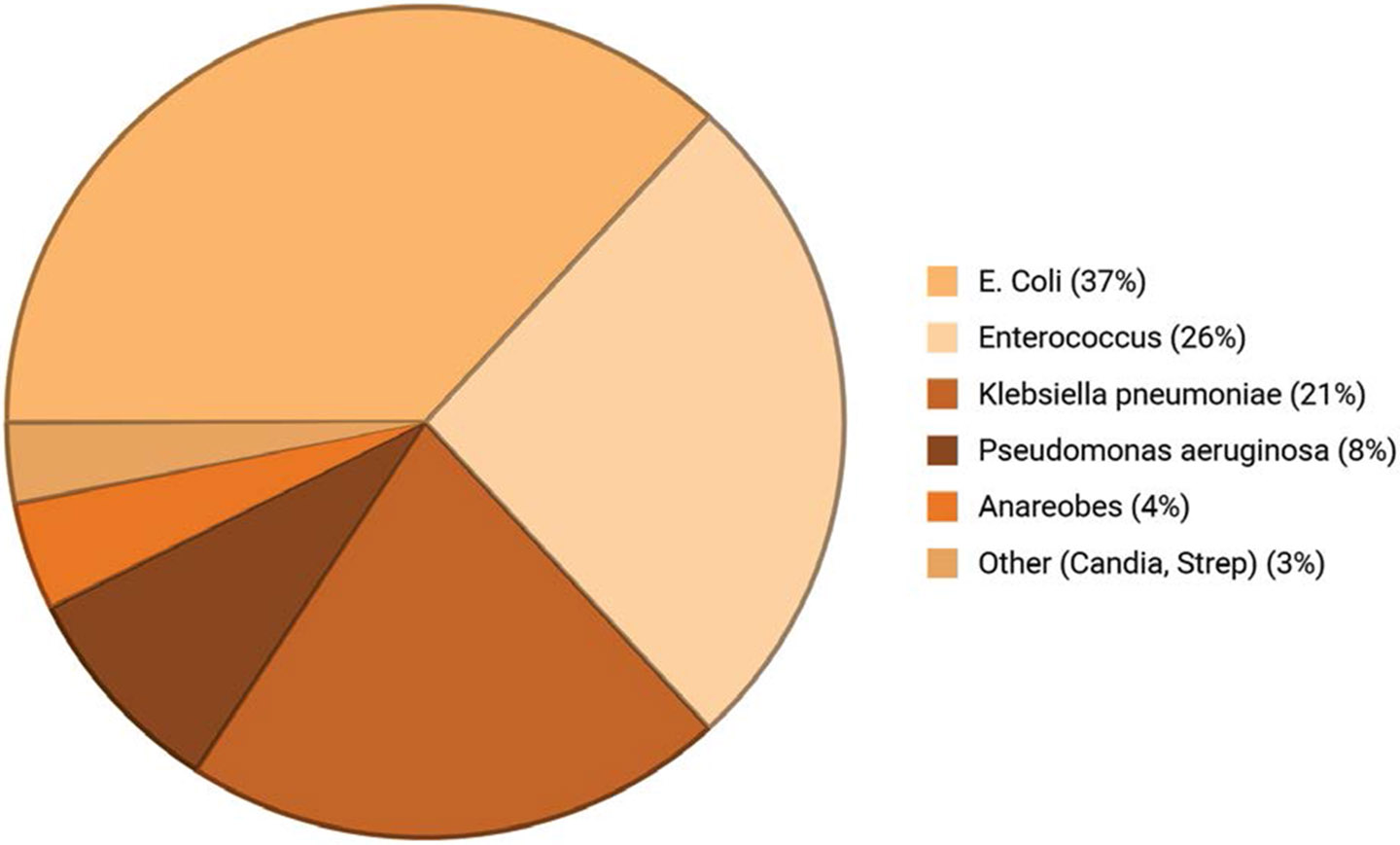
Commonly isolated infective agents in biliary tract infections. *Escherichia coli* represents the most frequent pathogen (37%), followed by *Enterococcus* species (26%) and *Klebsiella pneumoniae* (21%), with less frequent isolates including *Pseudomonas aeruginosa* (8%), anaerobes (4%), and other organisms such as *Candida* and *Streptococcus* species (3%). Based on averages and normalized.

Microbial colonization leads to complications through biofilm formation, altered bile acid composition, and local immune modulation, all of which impair healing. Postoperative infections commonly involve Enterococcus, Klebsiella, and Escherichia coli. Enterococcus faecalis and E. faecium are often linked with pancreatic fistulas and prior biliary drainage, while Klebsiella pneumoniae is frequently isolated in patients with biliary stenting [23]. (Figure 2).

Percutaneous biliary drainage (PBD) disrupts the sphincter of Oddi, allowing retrograde bacterial migration [24]. This frequently results in polymicrobial, multidrug-resistant infections, with E. coli colonization strongly associated with surgical site infections, abscesses, and pancreatic fistulas [25].

## Fungal and Anaerobic Coinfections

Fungal and anaerobic coinfections, especially after pancreaticoduodenectomy, further complicate management. Candida species are isolated in 17–50% of postoperative drainage cultures, with higher prevalence in patients receiving broad-spectrum antibiotics or biliary drainage [26]. Fungal infection correlates with higher-grade pancreatic fistula, major morbidity, hemorrhage, and surgical site infection, and Candida bloodstream infection can raise mortality to 69% in infected pancreatic necrosis [27].

## Multidrug Resistance and Antibiotic Management

**Figure 3:**
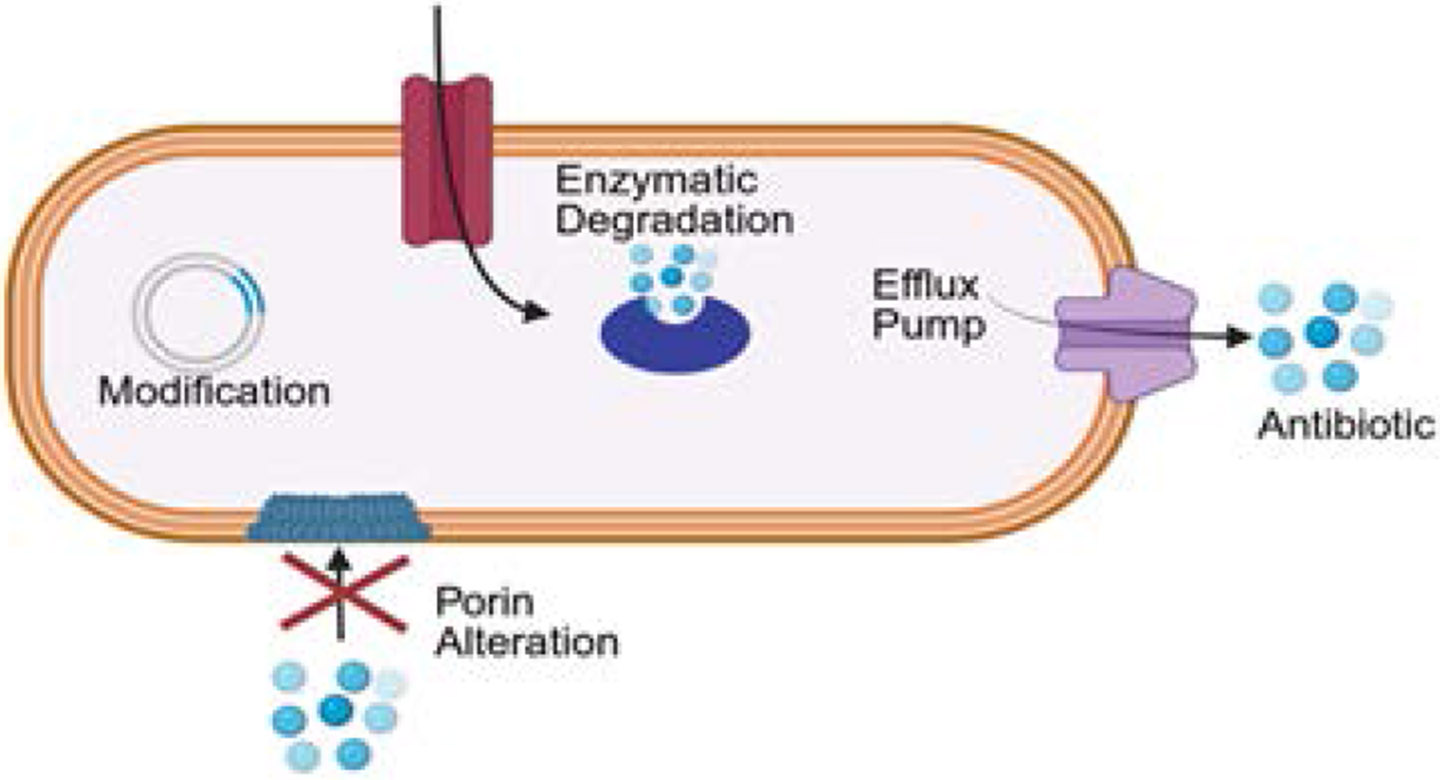
Representation of bacterial mechanisms of antibiotic resistance. Illustrated mechanisms include enzymatic degradation of antibiotics, activation of efflux pumps, alteration of membrane porins reducing drug uptake, and genetic modification conferring resistance traits.

Multidrug resistance (MDR) is a growing concern in postoperative infections involving bile contamination. Resistance arises primarily through enzymatic degradation of antibiotics, altered membrane permeability, efflux pump activation, and target site modification [28]. (Figure 3). Among Enterobacteriaceae, production of extended-spectrum β-lactamases (ESBLs) or AmpC β-lactamases confers resistance to penicillins and cephalosporins [29]. Some acquire carbapenemases such as New Delhi metallo-beta-lactamases (NDM) or OXA-type enzymes (oxacillinases), while Pseudomonas aeruginosa and Acinetobacter baumannii display resistance via efflux overexpression and biofilm tolerance [30].

Preoperative biliary drainage (PBD) further promotes resistance by disrupting the biliary barrier and introducing gut or hospital flora, which develop MDR through repeated antibiotic exposure [31]. Common MDR pathogens include Enterococcus spp., Klebsiella pneumoniae, and Escherichia coli. Bile stasis—resulting from obstruction or stent use—impairs bile flow and promotes bacterial overgrowth, leading to bacterobilia and polymicrobial colonization [32]. Biofilm formation on stents and drains worsens resistance by shielding bacteria from immune and antibiotic effects, often involving vancomycin-resistant Enterococcus and azoleresistant Candida [33].

Empirical broad-spectrum coverage (e.g., piperacillin-tazobactam) is recommended postoperatively, with therapy narrowed based on cultures. Duration depends on infection severity and source control—short courses for superficial infections, and 4–7 days for intra-abdominal or bloodstream infections [34]. Antifungal therapy is indicated for Candida infection or high-risk patients [35]. All treatment should follow antibiotic stewardship principles, emphasizing early initiation, targeted therapy, and avoidance of unnecessary prolonged use to prevent further resistance [36].

## Diagnosis of Multidrug-Resistant Infections

Diagnosis of multidrug-resistant (MDR) infections relies on clinical, laboratory, and microbiological assessments [37]. Laboratory markers—persistent leukocytosis, elevated C-reactive protein, and procalcitonin—suggest ongoing infection despite treatment, though they lack specificity for resistance mechanisms [38].

Bile, wound, and drain cultures provide the most direct evidence of resistance. Isolation of MDR organisms such as extended-spectrum β-lactamases-producing E. coli, Klebsiella pneumoniae, vancomycin-resistant Enterococcus, and carbapenem-resistant Enterobacter confirms resistance [39]. Antibiotic susceptibility testing identifies patterns—resistance to cephalosporins, fluoroquinolones, carbapenems, or glycopeptides—revealing underlying mechanisms like β-lactamase production, altered permeability, efflux activity, and target modification [40-42].

**Figure 4:**
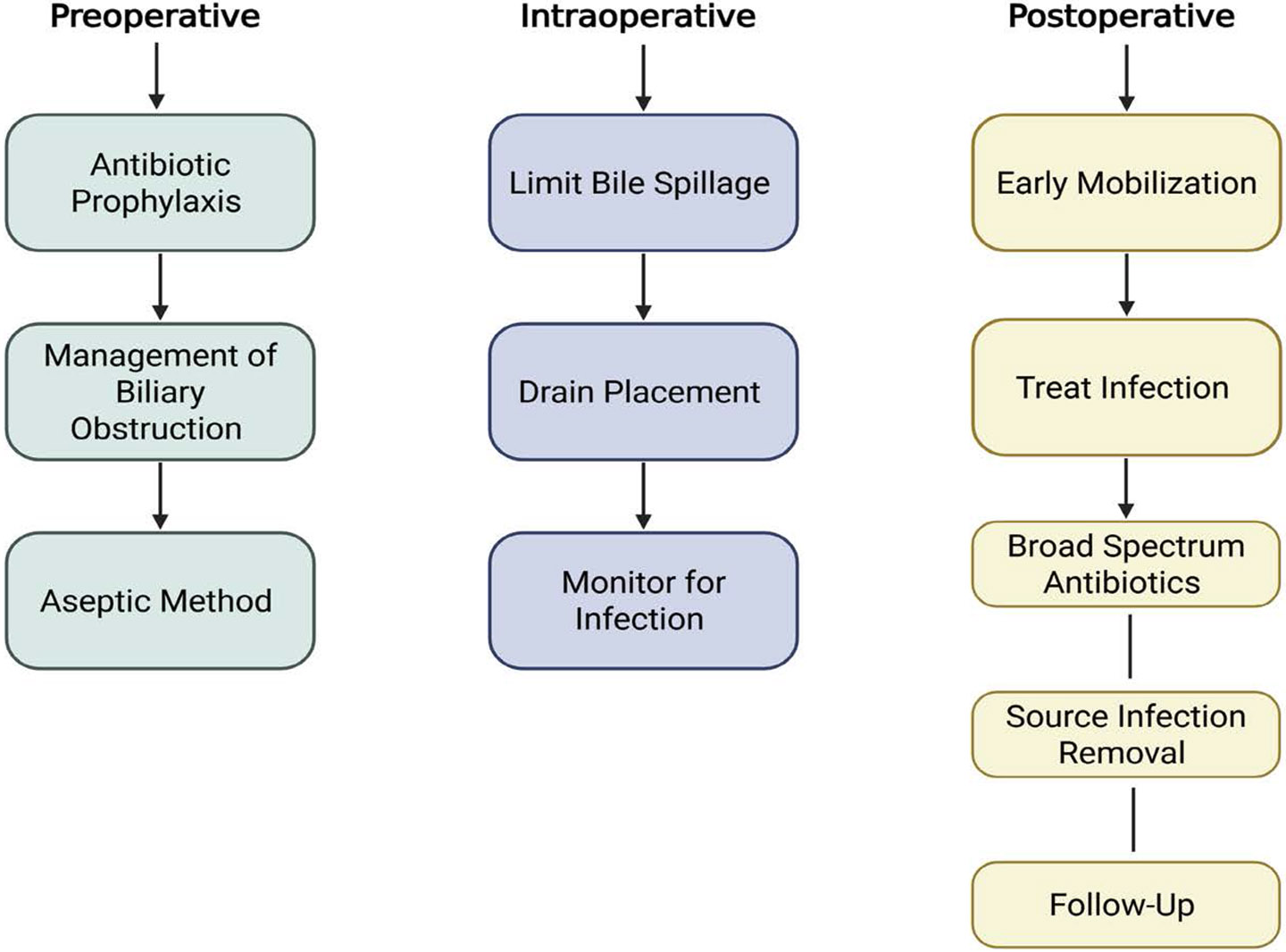
Approach to infection prevention and management in biliary surgery. Key strategies are shown across the perioperative continuum: preoperative (antibiotic prophylaxis, management of biliary obstruction, aseptic techniques), intraoperative (limiting bile spillage, drain placement, infection monitoring), and postoperative phases (early mobilization, infection treatment, broad-spectrum antibiotic therapy, source control, and follow-up).

Polymicrobial colonization is most common in patients undergoing biliary drainage and significantly increases the risk of postoperative infection [43]. Preoperative biliary drainage can alter bile sterility, leading to high rates of colonization with polymicrobial and drug-resistant organisms, including Enterobacteriaceae such as ESBL-producing Escherichia coli and Klebsiella pneumoniae, as well as Enterococcus species [44]. Multidrug-resistant (MDR) colonization is an independent risk factor for surgical site infections, postoperative pancreatic fistula, sepsis, and overall morbidity [45]. Furthermore, these organisms can form biofilms within biliary stents, which protect pathogens from both antibiotic activity and host immune responses [46] (Figure 4).

The identification of identical resistant organisms in both bile and wound or drain cultures confirms the pathogen source and enables targeted antimicrobial therapy [47]. Concordance is highest for Enterococcus spp., E. coli, K. pneumoniae, ESBL-producing Enterobacteriaceae, and vancomycin-resistant Enterococcus (VRE) [48]. This confirmation supports culture-guided adjustments to therapy, allowing for early de-escalation of broad-spectrum empiric antibiotics or escalation when resistance is identified [49]. While concordance rates are high, the predictive value of bile cultures for specific resistance profiles is limited by selective pressures during therapy and biofilm-mediated resistance [50].

Antimicrobial stewardship programs play a critical role in managing these infections by facilitating preoperative MDR screening, intraoperative bile sampling, and tailored prophylactic regimens [51]. Studies have demonstrated that integrating these strategies reduces postoperative infection rates in high-risk hepatobiliary and pancreatic surgeries [52].

Imaging modalities are essential for detecting abscesses, fluid collections, bile leaks, and anastomotic failures in patients with suspected infections [53]. In adults presenting with fever and sepsis, computed tomography is the first-line modality for identifying deep or complex intra-abdominal collections. Ultrasound is useful for rapid bedside assessment and percutaneous drainage of large collections but is limited in evaluating deeper or gas-containing lesions. Magnetic resonance imaging (MRI), particularly magnetic resonance cholangiopancreatography (MRCP), provides detailed delineation of biliary anatomy and detection of subtle leaks or ductal obstruction when other imaging modalities are inconclusive. Radiologic signs of infection include rimenhancing fluid collections, intralesional gas, and contrast extravasation at anastomotic sites [54,55].

The Infectious Diseases Society of America (IDSA) and the American College of Radiology (ACR) recommend integrating imaging and microbiologic findings for the management of complicated hepatobiliary and pancreatic infections. Imaging alone cannot distinguish sterile from infected collections, and intraoperative culture sampling is recommended when imaging identifies suspicious collections in symptomatic patients. The combination of radiologic evidence of abscess or leak with the isolation of MDR organisms from bile, wound, or drain fluid confirms the diagnosis and guides both source control—via drainage or reoperation—and targeted antimicrobial therapy, optimizing patient outcomes while limiting the spread of resistance [56-58].

## Clinical Presentation and Management

Patients with postoperative infections typically present with fever, leukocytosis, abdominal pain, wound erythema, purulent drainage, or dehiscence [59]. Severe infections may involve abscesses or anatomical leaks, requiring radiological drainage or surgical reintervention [60].

Management requires a multidisciplinary approach incorporating clinical assessment, imaging, microbiology, and source control [61]. Early empirical antibiotic therapy is essential, with piperacillin-tazobactam preferred in high-risk patients. Therapy is refined based on culture results, and antifungal coverage is added when indicated. Duration depends on infection severity and control success, guided by stewardship principles to prevent resistance [62-65].

## Future Directions and Conclusions

Postoperative infections continue to pose significant challenges in complex bile duct surgeries such as hepatectomy and pancreaticoduodenectomy. These complications stem from patient comorbidities, surgical complexity, perioperative factors, and biliary microbial colonization. Preoperative biliary interventions and obstruction increase the likelihood of polymicrobial, multidrug-resistant infections involving E. coli, Klebsiella pneumoniae, and Enterococcus species.

Despite improvements in surgical techniques and perioperative care, infections contribute to major morbidity, extended hospitalization, and increased costs. Future efforts should aim to optimize perioperative management, refine the indications and timing of biliary drainage, and improve understanding of microbial colonization and resistance evolution. Enhanced recovery strategies—including minimally invasive techniques, nutritional optimization, glycemic control, and immune modulation—along with rapid diagnostics and precision antimicrobial therapy, may enable personalized patient care. Integrating predictive models based on patient- and procedure-specific risk factors will be essential to reduce morbidity, improve outcomes, and establish evidence-based protocols for infection prevention and management in hepatobiliary surgery.

## Key Points

- Hepatectomy and pancreaticoduodenectomy are associated with postoperative infection rates ranging from 24% to 61%. This is due to surgical complexity, patient comorbidities, and perioperative factors such as blood loss and prolonged operative time.
- Preoperative biliary drainage, bile stasis, and biofilm formation on stents and drains significantly promote the risk of polymicrobial and multidrug-resistant (MDR) infections, most caused by Escherichia coli, Klebsiella pneumoniae, and Enterococcus species.
- Resistance mechanisms include β-lactamase production, efflux pump activity, altered membrane permeability, and target site modification. These are becoming increasingly prevalent, leading to more complicated postoperative management and antibiotic selection.
- Diagnosis of infections relies on clinical, laboratory, and microbiological evidence, with culture and susceptibility testing guiding targeted therapy. Early empirical broad-spectrum antibiotics, followed by de-escalation and strict antimicrobial stewardship, are key to controlling resistance.
- Optimization of perioperative management, including tailored antibiotic prophylaxis, nutritional support, minimally invasive approaches, and rapid diagnostics, holds promise for reducing infection rates and improving outcomes in hepatobiliary surgery.

## References

[1] Mentor K, Ratnayake B, Akter N, Meta-Analysis and Meta-Regression of Risk Factors for Surgical Site Infections in Hepatic and Pancreatic Resection. World J Surg 44 (2020): 4221–4230. 32812136 10.1007/s00268-020-05741-6

[2] Bone M, Latimer S, Walker RM, Risk factors for surgical site infections following hepatobiliary surgery: An umbrella review and meta-analyses. Eur J Surg Oncol 51 (2025): 109468. 39579465 10.1016/j.ejso.2024.109468

[3] Isik O, Kaya E, Sarkut P, Factors Affecting Surgical Site Infection Rates in Hepatobiliary Surgery. Surg Infect (Larchmt) 16 (2015): 281–286. 25830815 10.1089/sur.2013.195

[4] Sudo T, Murakami Y, Uemura K, Specific antibiotic prophylaxis based on bile cultures is required to prevent postoperative infectious complications in pancreatoduodenectomy patients who have undergone preoperative biliary drainage. World J Surg 31 (2007): 2230–2235. 17726628 10.1007/s00268-007-9210-4

[5] Shen Y, Hu YL, Xu JH, Incidence, risk factors, outcomes, and prediction model of surgical site infection after hepatectomy for hepatocellular carcinoma: A multicenter cohort study. Eur J Surg Oncol 51 (2025): 109486. 39615293 10.1016/j.ejso.2024.109486

[6] Noorian S, Kwaan MR, Jaffe N, Perioperative nutrition for gastrointestinal surgery: On the cutting edge. Nutr Clin Pract 38 (2023): 539–556. 36847684 10.1002/ncp.10970

[7] Khan A, Wong J, Riedel B, The Impact of Perioperative Enteral Immunonutrition on Post-operative Complications in Gastrointestinal Cancer Surgery: A Meta-Analysis. Ann Surg Oncol 30 (2023): 3619–3631. 36820938 10.1245/s10434-023-13265-1

[8] PancreasGroup.org Collaborative. Pancreatic surgery outcomes: multicentre prospective snapshot study in 67 countries. Br J Surg 111 (2024): 330. 10.1093/bjs/znad330 PMC10771125 38743040

[9] Tran TB, Dua MM, Spain DA, Hepatopancreatectomy: how morbid? Results from the national surgical quality improvement project. HPB (Oxford) 17 (2015): 763–769. 26058463 10.1111/hpb.12426 PMC4557649

[10] Lim C, Ishizawa T, Miyata A, Surgical Indications and Procedures for Resection of Hepatic Malignancies Confined to Segment VII. Ann Surg 263 (2016): 529–537. 25563884 10.1097/SLA.0000000000001118

[11] Lau WY, Lai EC, Lau SH. Management of bile duct injury after laparoscopic cholecystectomy: a review. ANZ J Surg 80 (2010): 75–81. 20575884 10.1111/j.1445-2197.2009.05205.x

[12] Lim C, Ishizawa T, Miyata A, Surgical Indications and Procedures for Resection of Hepatic Malignancies Confined to Segment VII. Ann Surg 263 (2016): 529–537. 25563884 10.1097/SLA.0000000000001118

[13] Benson AB, D'Angelica MI, Abbott DE, Hepatobiliary Cancers, Version 2.2021, NCCN Clinical Practice Guidelines in Oncology. J Natl Compr Canc Netw 19 (2021): 541–565. 34030131 10.6004/jnccn.2021.0022

[14] Shubert CR, Habermann EB, Truty MJ, Defining perioperative risk after hepatectomy based on diagnosis and extent of resection. J Gastrointest Surg 18 (2014): 1917–1928. 25199947 10.1007/s11605-014-2634-x

[15] Brown ZJ, Tsilimigras DI, Ruff SM, Management of Hepatocellular Carcinoma: A Review. JAMA Surg 158 (2023): 410–420. 36790767 10.1001/jamasurg.2022.7989

[16] Donahue TR, Reber HA. Surgical management of pancreatic cancer--pancreaticoduodenectomy. Semin Oncol 42 (2015): 98–109. 25726055 10.1053/j.seminoncol.2014.12.009

[17] Pappas S, Krzywda E, McDowell N. Nutrition and pancreaticoduodenectomy. Nutr Clin Pract 30 (2015): 162. 20581316 10.1177/0884533610368709

[18] Mihaljevic AL, Hackert T, Loos M, Not all Whipple procedures are equal: Proposal for a classification of pancreatoduodenectomies. Surgery 169 (2021): 1456–1462. 33386130 10.1016/j.surg.2020.11.030

[19] Van der Pol CB, Sabil M, Komar M, Society of Abdominal Radiology Pancreatic Ductal Adenocarcinoma Disease Focus Panel. Factors Associated with Aborted Whipple Procedures for Periampullary Carcinoma: A Multicenter Case-Control Study by the SAR Pancreatic Ductal Adenocarcinoma Disease Focus Panel. AJR Am J Roentgenol 224 (2025): e2432160. 40042924 10.2214/AJR.24.32160

[20] Mangieri CW, Strode MA, Valenzuela CD, High-risk liver patients are not associated with adverse events following pancreaticoduodenectomy. Am J Surg 225 (2023): 735–739. 36428108 10.1016/j.amjsurg.2022.11.007

[21] Tamburrino D, Guarneri G, Provinciali L, Effect of preoperative biliary stent on postoperative complications after pancreaticoduodenectomy for cancer: Neoadjuvant versus upfront treatment. Surgery 172 (2022): 1807–1815. 36253311 10.1016/j.surg.2022.09.001

[22] Olecki EJ, Swinarska J, Perez Holguin RA, Is preoperative biliary stenting associated with increased rate of postoperative complications for patients undergoing pancreatoduodenectomy? A review of national surgical quality improvement program data. HPB (Oxford) 24 (2022): 1501–1510. 35135722 10.1016/j.hpb.2022.01.006

[23] Scheufele F, Aichinger L, Jäger C, Effect of preoperative biliary drainage on bacterial flora in bile of patients with periampullary cancer. Br J Surg 104 (2017): e182–e188. 28121036 10.1002/bjs.10450

[24] Asukai K, Akita H, Mukai Y, The utility of bile juice culture analysis for the management of postoperative infection after pancreaticoduodenectomy. Surgery 173 (2023): 1039–1044. 36549976 10.1016/j.surg.2022.11.021

[25] Kimura N, Ishido K, Wakiya T, Revealing the role of early peripancreatic bacterial contamination and Enterococcus faecalis in pancreatic fistula development after pancreaticoduodenectomy: Implications for useful antibiotic prophylaxis-An observational cohort study. Pancreatology 24 (2024): 630–642. 38508910 10.1016/j.pan.2024.03.008

[26] Abe K, Kitago M, Shinoda M, High risk pathogens and risk factors for postoperative pancreatic fistula after pancreatectomy; a retrospective case-controlled study. Int J Surg 82 (2020):136–142. 32861892 10.1016/j.ijsu.2020.08.035

[27] Behrman SW, Bahr MH, Dickson PV, The microbiology of secondary and postoperative pancreatic infections: implications for antimicrobial management. Arch Surg 146 (2011): 613–619. 21576614 10.1001/archsurg.2011.85

[28] Tamburrino D, Guarneri G, Provinciali L, Multidrug-resistant bacterial colonization affects postoperative outcomes after pancreaticoduodenectomy. Surgery 186 (2025): 109594. 40780055 10.1016/j.surg.2025.109594

[29] Gibiino G, Cucchetti A, Mocchegiani F, Alarming correlation between multidrug-resistant bacteriobilia and morbidity after pancreatic surgery. Dig Liver Dis 55 (2023): 1502–1508. 37263811 10.1016/j.dld.2023.05.012

[30] Yang Y, Duan Y, Su C, The impact of preoperative biliary drainage on bile colonization of patients undergoing pancreaticoduodenectomy. Ann Med. 2025 Dec;57(1):2540024. 40742353 10.1080/07853890.2025.2540024 PMC12315152

[31] Bush K, Bradford PA. Epidemiology of β-Lactamase-Producing Pathogens. Clin Microbiol Rev 33 (2020): e00047–19. 32102899 10.1128/CMR.00047-19 PMC7048014

[32] Macesic N, Uhlemann AC, Peleg AY. Multidrug-resistant Gram-negative bacterial infections. Lancet 405 (2025): 257–272. 39826970 10.1016/S0140-6736(24)02081-6

[33] Perez F, El Chakhtoura NG, Bonomo RA. Management of Severe Infections: Multidrug-Resistant and Carbapenem-Resistant Gram-Negative Bacteria. Med Clin North Am 109 (2025): 735–747. 40185559 10.1016/j.mcna.2025.01.003

[34] Giannone F, Lagarrigue C, Ligurgo O, Adaptation of antibiotics and antifungal strategy to preoperative biliary drainage to improve postoperative outcomes after pancreatic head resection. World J Surg (2025): 270–282. 10.1002/wjs.12446 PMC11711118 39681975

[35] D'Angelica MI, Ellis RJ, Liu JB, Piperacillin-Tazobactam Compared With Cefoxitin as Antimicrobial Prophylaxis for Pancreatoduodenectomy: A Randomized Clinical Trial. JAMA 329 (2023): 1579–1588. 37078771 10.1001/jama.2023.5728 PMC10119777

[36] Noll J, Roosen H, Fritzenwanker M, Perioperative multidrug-resistant bacteria impair clinical outcome after pancreatic surgery: Missed targets of antibiotic prophylaxis. Surgery 188 (2025): 109717. 41061330 10.1016/j.surg.2025.109717

[37] Liu T, Li M, Tang L, Epidemiological, clinical and microbiological characteristics of patients with biliary tract diseases with positive bile culture in a tertiary hospital. BMC Infect Dis 24 (2024): 1010. 39300331 10.1186/s12879-024-09799-8 PMC11414084

[38] Chen S, Lai W, Song X, The distribution and antibiotic-resistant characteristics and risk factors of pathogens associated with clinical biliary tract infection in humans. Front Microbiol 15 (2024): 1404366. 38784792 10.3389/fmicb.2024.1404366 PMC11112516

[39] Gu XX, Zhang MP, Zhao YF, Clinical and microbiological characteristics of patients with biliary disease. World J Gastroenterol 26 (2020): 1638–1646. 32327912 10.3748/wjg.v26.i14.1638 PMC7167412

[40] Macesic N, Uhlemann AC, Peleg AY. Multidrug-resistant Gram-negative bacterial infections. Lancet 405 (2025): 257–272. 39826970 10.1016/S0140-6736(24)02081-6

[41] Simner PJ, Pitout JDD, Dingle TC. Laboratory detection of carbapenemases among Gram-negative organisms. Clin Microbiol Rev 37 (2024): e0005422. 39545731 10.1128/cmr.00054-22 PMC11629623

[42] Stathopoulos P, Lerner P, Astheimer P, Endoscopic retrograde cholangiopancreatography-obtained bile culture in acute cholangitis: retrospective analysis of bile cultures and risk factors in a tertiary care center. J Gastroenterol Hepatol 39 (2024): 935–941. 38267213 10.1111/jgh.16492

[43] Yang Y, Duan Y, Su C, The impact of preoperative biliary drainage on bile colonization of patients undergoing pancreaticoduodenectomy. Ann Med 57 (2025): 2540024. 40742353 10.1080/07853890.2025.2540024 PMC12315152

[44] Sudo T, Murakami Y, Uemura K, Specific antibiotic prophylaxis based on bile cultures is required to prevent postoperative infectious complications in pancreatoduodenectomy patients who have undergone preoperative biliary drainage. World J Surg 31 (2007): 2230–2235. 17726628 10.1007/s00268-007-9210-4

[45] Tamburrino D, Guarneri G, Provinciali L, Multidrug-resistant bacterial colonization affects postoperative outcomes after pancreaticoduodenectomy. Surgery 186 (2025): 109594. 40780055 10.1016/j.surg.2025.109594

[46] Windisch O, Frossard JL, Schiffer E, Microbiologic Changes Induced by Biliary Drainage Require Adapted Antibiotic Prophylaxis during Duodenopancreatectomy. Surg Infect (Larchmt) 20 (2019): 677–682. 31298622 10.1089/sur.2019.088

[47] Herzog T, Belyaev O, Akkuzu R, The Impact of Bile Duct Cultures on Surgical Site Infections in Pancreatic Surgery. Surg Infect (Larchmt) 16 (2015): 443–449. 26110464 10.1089/sur.2014.104

[48] Asukai K, Akita H, Mukai Y, The utility of bile juice culture analysis for the management of postoperative infection after pancreaticoduodenectomy. Surgery 173 (2023): 1039–1044. 36549976 10.1016/j.surg.2022.11.021

[49] Mohan A, Gupta R, Yadav TD, Association of Intra-Operative Bile Culture with Post-Operative Complications after Pancreaticoduodenectomy. Surg Infect (Larchmt) 23 (2022): 351–356. 35231198 10.1089/sur.2021.215

[50] Maxwell DW, Jajja MR, Ferez-Pinzon A, Bile cultures are poor predictors of antibiotic resistance in postoperative infections following pancreaticoduodenectomy. HPB (Oxford) 22 (2020): 969–978. 31662223 10.1016/j.hpb.2019.10.016

[51] De Pastena M, Paiella S, Lionetto G, An Antimicrobial Stewardship Program in Pancreatic Surgery Reduces the Infectious Risk of Colonized Bile, Reducing the Predictive Value of the Intraoperative Bile Culture: A Before-after Study on 1638 Pancreatoduodenectomies. Ann Surg 282 (2025): 725–733. 40747933 10.1097/SLA.0000000000006870

[52] De Pastena M, Paiella S, Secchettin E, An Antibiotic Stewardship Program in Pancreatic Surgery. JAMA Netw Open 8 (2025): e2520149. 40643910 10.1001/jamanetworkopen.2025.20149 PMC12254888

[53] Bonomo RA, Chow AW, Edwards MS, 2024 Clinical Practice Guideline Update by the Infectious Diseases Society of America on Complicated Intra-abdominal Infections: Risk Assessment, Diagnostic Imaging, and Microbiological Evaluation in Adults, Children, and Pregnant People. Clin Infect Dis 79 (2024): S81–S87. 38965057 10.1093/cid/ciae346

[54] Huston JM, Barie PS, Dellinger EP, Therapeutics and Guidelines Committee. The Surgical Infection Society Guidelines on the Management of Intra-Abdominal Infection: 2024 Update. Surg Infect (Larchmt) 25 (2024): 419–435. 38990709 10.1089/sur.2024.137

[55] Nair RT, Chan A, Morgan MA, Biliary complications of surgical procedures: what the radiologist needs to know. Abdom Radiol (NY) 50 (2025): 2999–3019. 39738660 10.1007/s00261-024-04754-2

[56] Bilgiç Ç, Keske Ş, Sobutay E, Surgical site infections after pancreaticoduodenectomy: Preoperative biliary system interventions and antimicrobial prophylaxis. Int J Infect Dis 95 (2020): 148–152. 32278107 10.1016/j.ijid.2020.04.005

[57] Pham H, Chen A, Nahm CB, The Role of Targeted Versus Standard Antibiotic Prophylaxis in Pancreatoduodenectomy in Reducing Postoperative Infectious Complications: A Systematic Review and Meta-analysis. Ann Surg 275 (2022): 315–323. 33630442 10.1097/SLA.0000000000004816

[58] Asukai K, Akita H, Mukai Y, The utility of bile juice culture analysis for the management of post-operative infection after pancreaticoduodenectomy. Surgery 173 (2023): 1039–1044. 36549976 10.1016/j.surg.2022.11.021

[59] Simon R. Complications After Pancreaticoduodenectomy. Surg Clin North Am 101 (2021): 865–874. 34537148 10.1016/j.suc.2021.06.011

[60] Jin S, Fu Q, Wuyun G, Management of post-hepatectomy complications. World J Gastroenterol (2013): 7983–7991. 24307791 10.3748/wjg.v19.i44.7983 PMC3848145

[61] Gaballah AH, Kazi IA, Zaheer A, Imaging after Pancreatic Surgery: Expected Findings and Postoperative Complications. Radiographics 44 (2024): e230061. 38060424 10.1148/rg.230061

[62] Okano K, Hirao T, Unno M, Postoperative infectious complications after pancreatic resection. Br J Surg 102 (2015): 1551–160. 26387569 10.1002/bjs.9919

[63] Asukai K, Akita H, Mukai Y, The utility of bile juice culture analysis for the management of postoperative infection after pancreaticoduodenectomy. Surgery 173 (2023): 1039–1044. 36549976 10.1016/j.surg.2022.11.021

[64] Chen X, Sun S, Yan X, Predictive Factors and Microbial Spectrum for Infectious Complications after Hepatectomy with Cholangiojejunostomy in Perihilar Cholangiocarcinoma. Surg Infect (Larchmt) 21 (2020): 275–283. 31710266 10.1089/sur.2019.199

[65] Gaballah AH, Kazi IA, Zaheer A, Imaging after Pancreatic Surgery: Expected Findings and Postoperative Complications. Radiographics 44 (2024): e230061. 38060424 10.1148/rg.230061

